# Low-Cost System Based on Optical Sensor to Monitor Discharge of Industrial Oil in Irrigation Ditches

**DOI:** 10.3390/s21165449

**Published:** 2021-08-12

**Authors:** Daniel A. Basterrechea, Javier Rocher, Lorena Parra, Jaime Lloret

**Affiliations:** 1 Instituto de Investigación para la Gestión Integrada de Zonas Costeras, Universitat Politècnica de València, C/Paraninf, 1 Grao de Gandia, 46730 Valencia, Spain; dabasche@epsg.upv.es (D.A.B.); jarocmo@doctor.upv.es (J.R.); loparbo@doctor.upv.es (L.P.); 2 IMIDRA, Finca “El Encin”, A-2, Km 38, 2 Alcalá de Henares, 28805 Madrid, Spain

**Keywords:** oil sensor, smart farming, irrigation channels, light absorption, engine oil, LED, photosensor, illicit discharge

## Abstract

Uncontrolled dumping linked to agricultural vehicles causes an increase in the incorporation of oils into the irrigation system. In this paper, we propose a system based on an optical sensor to monitor oil concentration in the irrigation ditches. Our prototype is based on the absorption and dispersion of light. As a light source, we use Light Emitting Diodes (LEDs) with different colours (white, yellow, blue, green, and red) and a photodetector as a sensing element. To test the sensor’s performance, we incorporate industrial oils used by a diesel or gasoline engine, with a concentration from 0 to 0.20 mL_oil_/cm^2^. The experiment was carried out at different water column heights, 0 to 20 cm. According to our results, the sensor can differentiate between the presence or absence of diesel engine oil with any LED. For gasoline engine oil, the sensor quantifies its concentration using the red light source; concentrations greater than 0.1 mL_oil_/cm^2^ cannot be distinguished. The data gathered using the red LED has an average absolute error of 0.003 mL_oil_/cm^2^ (relative error of 15.8%) for the worst case, 15 cm. Finally, the blue LED generates different signals in the photodetector according to the type of oil. We developed an algorithm that combines (i) the white LED, to monitor the presence of oil; (ii) the blue LED, to identify if the oil comes from a gasoline or diesel engine; and (iii) the red LED, to monitor the concentration of oil used by a gasoline engine.

## 1. Introduction

The demand for food has increased drastically in recent years due to population growth and the changes in society’s lifestyle [1,2]. In this context, farmers have been forced to increase their productivity. In order to increase it, farmers resort to different ways, either through the incorporation of new technologies or through the use of farm machinery, fertilizers, pesticides, etc.

Nonetheless, the incorporation of new types of machinery can cause problems in the environment. One of these problems is the use of oil for their operation. Due to accidents or negligence, the oil from the engine can reach the irrigation channel. The industry is another source of pollution; some industries can incorporate used oil into the water by illegal dumping. These discharges often end up in different water bodies such as channels or ditches, whose water can be used for irrigation purposes. The polluted water used for irrigation can infiltrate and pollute the soil, the crops, or the groundwater or can be diluted into larger water bodies (rivers, reservoirs, sea, etc.) The most common water sources used in irrigation are groundwater and irrigation ditches [3].

Oil-polluted water represents a danger to the crop’s quality or safety and the farmers’ economy [4]. Due to the use of oil-polluted water, farmers suffer a significant reduction in the quality and quantity of their yield. This situation leads to income decrease and economic decline in the agricultural sector. Furthermore, oil can impact both present and future plantations because it is accumulated in the soil. The oil combined with the nutrients in the soil causes a reduction in the availability of nutrients for plants. In addition, the accumulation of oil in soil reduces the microorganism presence, eliminating the nitrogen-fixing bacteria. These bacteria are essential and have been proven to be a fundamental part of obtaining greater quality and quantity of crops.

Public institutions work to prevent illicit discharges. If an illicit discharge is detected, the authorities should investigate the case and report the obtained information to the public institution and sanction the responsible person. A solution to reduce illicit dumping into the environment is increasing the control methods [5]. However, the current type of control and detection is inefficient because surveillance is usually minimal if there is no suspicion of a possible contamination source. Moreover, investigation often requires a long time to complete, and the oil would have already been incorporated into the soil of the fields through irrigation, and removing the pollution from the soil is costly and ineffectual. Therefore, the polluter pays principle is not fulfilled. Other used methods for monitoring consist of taking samples of the irrigation water and sending them to the laboratory for chemical analysis [6]. These procedures are expensive because they require one person to take the samples and procure an external laboratory [7]. Thus, the existing methods are not suitable for continuous monitoring of irrigation water quality. For these reasons, in recent years, the use of Wireless Sensor Networks (WSNs) has increased to apply continuous control over the water quality. The WSN allows obtaining water quality data at a lower cost than the aforementioned chemical analysis in a laboratory.

In this paper, we propose a reflection-absorption sensor for monitoring the amount of industrial oil (used by a gasoline or diesel engine) present in water used for irrigation. For this purpose, we use industrial oil coming from gasoline and diesel engines as a pollution source. We selected 5 Light-Emitting Diodes (LED) with different colours (green, red, yellow, white, and blue) as light sources or signals. As a light receptor or sensor, we used Light-Dependent Resistors (LDRs). The light detectors were placed at 180° and 360° at different water column heights (5, 10, 15, and 20 cm). The operation of the LDR is based on the fact that the resistance decreases when exposure to light increases. These changes in its resistance will be measured when the sensor is exposed to different levels of pollution.

The rest of the paper is structured as follows. First, the related work is outlined in Section 2. Then, Section 3 explains the proposal of the monitoring system. Then the followed methodology and used materials are described in Section 4. Next, the results are presented in Section 5. Next, the implications of our results are discussed in Section 6. Finally, Section 7 summarizes the obtained conclusions.

## 2. Related Work

In this section, we present the current efforts in water quality monitoring based on the contribution of other authors. Once the proposals of different authors have been analysed, the advantages and improvements of the developed sensor will be highlighted. The related work is structured into two subsections. Section 2.1 summarizes the oil detection using remote sensing data. Then, Section 2.2 focuses on sensors for water quality monitoring.

### 2.1. Oil Detection Using Satellite Information

This subsection compares the different methods to detect and quantify oil in water using remote sensing data based on satellite information sources.

Peterson et al. [8] proposed the use of an airborne visible and infrared imaging spectrometer applying multiple endmember spectral mixture analysis to detect the presence of oil in the marshes of Barataria Bay (Louisiana, EEUU). The proposed system is based on the similarities in oil and non-photosynthetic vegetation (NPV) spectra, differing only in two narrow hydrocarbon absorption regions around 1700 nm and 2300 nm. Confusion between NPV and oil is expressed as an increase in oil fraction error with increasing NPV. The presented study obtained an accuracy ranging from 87.5% to 93.3% in oil detection. Another example of remote sensing is presented by Nezhad et al. [9] They proposed an oil spill detection system by analysing Sentinel 2 images in the Persian Gulf. Their study followed the evolution of one oil spill event using multi-sensor satellite images in the Al Khafji zone (between Kuwait and Saudi Arabia). Oil slicks were characterized with multi-sensor satellite images over the Persian Gulf. They analysed the bands in order to detect and classify oil spills in this zone. They used ENVI software for analysing satellite images and automated data inquiry for oil spills for oil weathering modelling. In addition, Taravat et al. [10] presented a study in which they examine Landsat ETM+ images’ feasibility to detect oil spill pollution in the Gulf of Mexico. The authors concluded that the bands’ difference between 660 nm and 560 nm, and division at 825 nm and 560 nm, normalized by 480 nm provide the best result. Another remote sensing-based approach for oil detection is presented by Zhao et al. [11] They studied the spectral signature in the visible and infrared domain of oil slicks observed in shallow coastal waters of the Arabian Gulf. They used a Moderate Resolution Imaging Spectroradiometer (MODIS), medium resolution imaging spectrometer, and Landsat data. Images of the floating algae index and estimation of sea currents from hydrodynamic models supported the multi-sensor oil tracking technique. They pointed out that scenes with and without sun glint as a spectral signature of oil slicks in the optical domain depends upon the viewing geometry and the solar angle. Depending on the combination of those factors, oil slicks may exhibit dark or bright contrasts concerning oil-free waters. To track oil slicks and forecast their potential landfall, they used ocean circulation and wind data.

Another alternative to detect oil spills was showed by Pisano et al. [12] They used 11 MODIS images, seven of them to develop the methodology and four for validating the method. The ratio image R = L′GN/LGN is used to detect possible areas with oil spills. In this ratio, L′GN is the MODIS-retrieved normalized sun glint radiance. The other variable LGN is an image correction independent of the wind direction obtained by Cox and Munk isotropic sun glint model. Their results indicated that using a threshold is possible to isolate the spills. As a result, the spills of the validation dataset are successfully detected.

Salberg et al. [13] examined the Specific Absorption Rate (SAR) of images operating in a hybrid-polarimetric mode to detect oil spills. They proposed and reviewed several strategies for oil spill detection based on hybrid-polarimetric SAR data. Their results pointed out that low-wind lookalikes may be suppressed simultaneously as the contrast of the oil spills is maintained using hybrid-polarimetry data. Thus, those multi-feature images may be constructed to enhance the performance of oil spill detection.

The methodologies previously presented are not a viable option for detecting oil in the irrigation system. This is because the irrigation channels are quite small, and most of the remote sensing data sources have spatial resolutions suitable only for larger areas. Moreover, the return time, or temporal resolutions, of satellites precludes the continuous monitoring of water. Therefore, another methodology must be applied for continuous monitoring of oil spills in small water bodies such as irrigation channels. The WSN with specific sensors for water quality can be a promising solution for these cases.

### 2.2. Sensors for Water Quality Monitoring

To analyse the available options in WSN, we focus this subsection on the current prototypes based on sensors for water quality.

Ding et al. [14] presented a review of the development of nanomaterial-based optical sensors for Hg^2+^ detection, which showed the benefits of simplicity, rapidity, high sensitivity, selectivity, and cost-effectiveness. They summarised the published innovations in nanomaterial-based optical sensors for the detection of Hg^2+^ according to different sensing strategies, including colourimetric, fluorescent, and surface-enhanced Raman scattering detection. Since used oil usually presents small concentrations of heavy metals, a method to detect used oil is through detecting those heavy metals. Although their proposal has several benefits, not all oil spills will have the concentration of Hg^2+^ to be witness, and it might lead to a false-negative result. Moreover, a nanomaterial-based sensor might have several requirements in terms of maintenance or replacing elements. A turbidity sensor able to distinguish different types of turbidity was developed by Parra et al. [15] The sensor is based on the Beer–Lambert law, and it uses four LEDs, with different colours, as light sources. Their sensor was able to differentiate among different turbidity sources. They calibrate their sensor using *Isochrysis galbana, Tetraselmis chuii*, and sediment. The results indicated that it is possible to determine the turbidity levels using infrared light and to characterise the origin of that turbidity with the red light in some conditions. Although this sensor with the current calibrations and its algorithm cannot be applied for the detection of oil, its operational principle is applicable. Thus, the combination of light sources as LEDs with different colours and an algorithm is included in the design of our prototype.

Another approach was followed by Hübner et al. [16], who developed a novel method to determine compositions on the bimodal of biphasic system. They proposed a method based on mass balance. This balance correlates a multicomponent biphasic system with a heterogeneous composition with the positon phase interface in the microchannel. Finally, they proposed an extension of the method using the information on the bimodal compositions in a subsequent step to calculate phase equilibrium compositions. In the same context, Ramírez-Miquet et al. [17] proposed implementing optical feedback interferometry for the analysis at the microscale of multiphase flows, starting with the case of parallel flows of two immiscible fluids. This implementation will be a new tool for studying more complex interactions between immiscible fluids. Nonetheless, its application in irrigation channels might be challenging due to the heterogeneity in the suspended solids, which might cause problems with microfluidic technologies.

McCue et al. [18] proposed a new modular mid-infrared evanescent wave fibre optic sensor to detect hydrocarbons in water. The sensor uses a broadband source with back-reflecting optics coupled to a fibre optic sensing element. This is coated with an analyse-enriching polymer that concentrates the analyse in the sensing region. The experiment results indicated an optimal behaviour of the sensor, with a concentration of benzene below 500 ppm. Next, Oliver Péron et al. [19] presented an accurate synthesis of Surface-enhanced Raman scattering/spectroscopy active substrates, based on a gold colloidal monolayer, suitable for in situ environmental analysis. They demonstrated that Au-colloidal hydrophobic films synthesized by quartz salinization provide polycyclic aromatic hydrocarbon (PAH) pre-concentration as well as an SERS effect. Then, Jackson S. Albuquerque et al. [20] proposed using poly(dimethylsiloxane) to detect aromatic hydrocarbons in water using near-infrared spectroscopy. They could classify the water into distinct groups, contaminated by gasoline A (without ethanol), gasoline C (with 25% of anhydrous ethanol), or diesel fuel. Even though all these methods have a high accuracy and low detection ranges, they are costly and cannot be easily implemented in an WSN for irrigation channel monitoring.

The use of LEDs as a light source (or signal) to monitor the presence of oil, in general terms, is not a new issue. A survey of the use of chemical sensing devices is presented by Yeh et al. [21] The chemical sensors analysed integrated LEDs in the range of Ultraviolet, visible, and infrared (247–3800 nm). The studied sensors were used for monitoring toxins, heavy metals, environmental nutrients, toxic gases, biochemical compounds, and biohazard compounds. They confirmed that LEDs are good light sources in chemical sensors. Meanwhile, Parra et al. [22] developed a low-cost optic sensor capable of detecting hydrocarbons on the water surface. They use LEDs as a light emitter and an LDR as a receiver. They use as light sources different colours (violet, blue, green, orange, red, and white). The used samples were made of seawater and fuel. The results showed that white was the best LED. Their results pointed out that their prototype can be sued to detect that the presence of oil was optimal. However, it cannot quantify the hydrocarbon concentration. Despite the fact that their sensor was able to detect the presence of certain hydrocarbons, no information about their possible use to detect industrial oil is given. In addition, the limitation of not being able to quantify the pollution makes difficulties for the tracking.

The optical sensors presented are not optimal to monitor oil in irrigation channels because the technology used is not suitable for an WSN for continuous monitoring or cannot measure oil in the water column. In the case of the sensor developed by Parra et al. [22], it can detect oil in water but cannot quantify it. Furthermore, the low-cost optical sensor that we have developed quantifies the amount of oil present in the water, making it a preferable sensor for monitoring oil in irrigation water.

## 3. Proposal

In this section, we summarise the included elements in our proposal. First, we show the characteristics of the proposed prototype. Our prototype is based on photoreceptors and colour LEDs. Finally, an operational algorithm that integrates the sensor on a WSN is displayed. The algorithm sends an alarm to the people responsible for water control to inform them that oil is detected. The control of oil presence in water is critical to prevent the contamination of soils. The proposed sensor is part of the SMARTWATIR project. This project aims to develop and deploy a WSN to detect and purify irrigation water in the Mediterranean area.

### 3.1. Description of the Optical System

**Figure 1:**
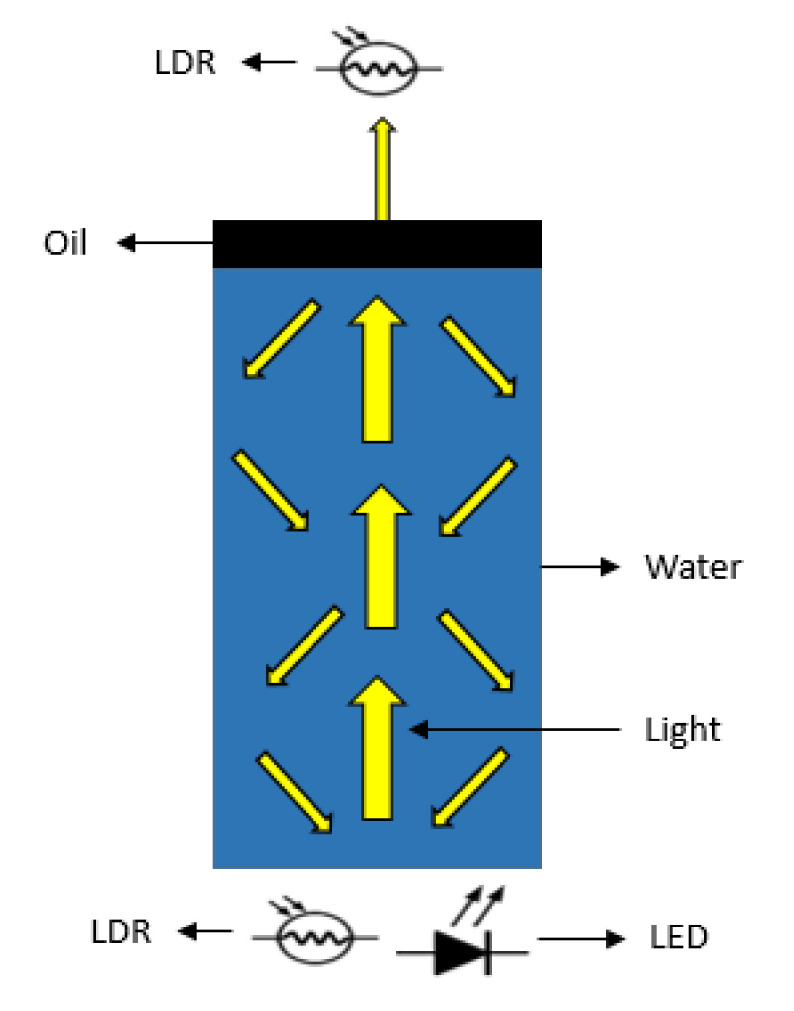
Sensor working diagram.

In our prototype, the light source is activated using five colour LEDs: blue, yellow, red, green, and white. Besides, photodetectors are selected to receive the amount of light absorbed, dispersed, reflected or passed through the water column. For this purpose, two photodetectors (LDRs) are located at 0° and 180° from the LEDs. We assume that the amount of the oil modifies the percentages of dispersed, reflected, absorbed or passed light, causing different lectures of the LDRs. The light radiated by the LEDs crosses the water column and hits the oil layer (if there is oil pollution). In cases where there is an oil layer, part of the light is reflected in the oil layer, going back to the LDR located at 0°, and another part crosses it, reaching the LDR located at 180°. The physical operating principle related to light dispersion and the location of LDRs and LEDs of the proposed sensor can be seen in Figure 1.

**Figure 2:**
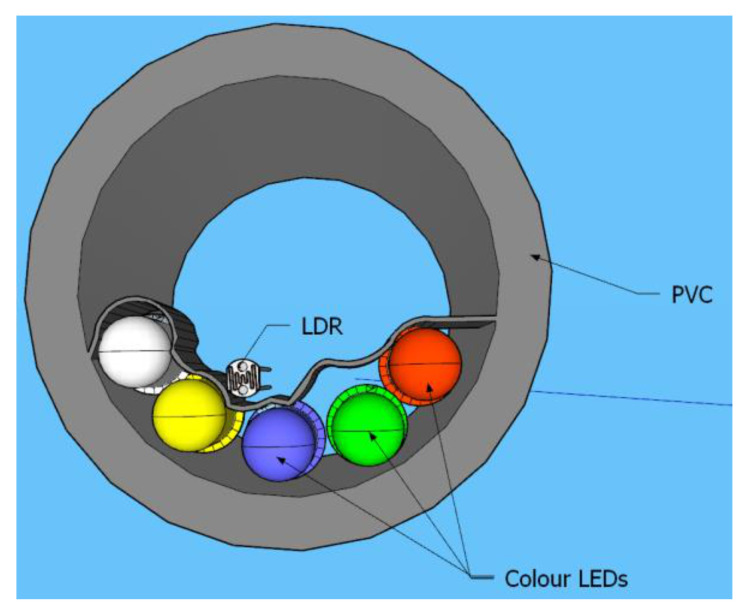
Design prototype.

The selected LEDs for our prototype are diffused LEDs with different function voltages. The function voltages are 1.8–2 V for yellow and red and 3–3.4 V for white, blue, and green LEDs. The vision angle is 20°, the maximum recommended intensity is 20 mA for all of them. As a photodetector, the NSL-19M51 (5 mm) was selected. Finally, the prototype is crafted with a PVC pipe characterized by an external and internal diameter of 5 cm and 4 cm, with a thickness of 5 mm. Figure 2 depicts the preliminary design of the proposed prototype. To test this prototype’s usability and obtain a calibration, we placed the prototype below a glass tank with water, and we add different amounts of oil.

### 3.2. Operational Algorithm

**Figure 3:**
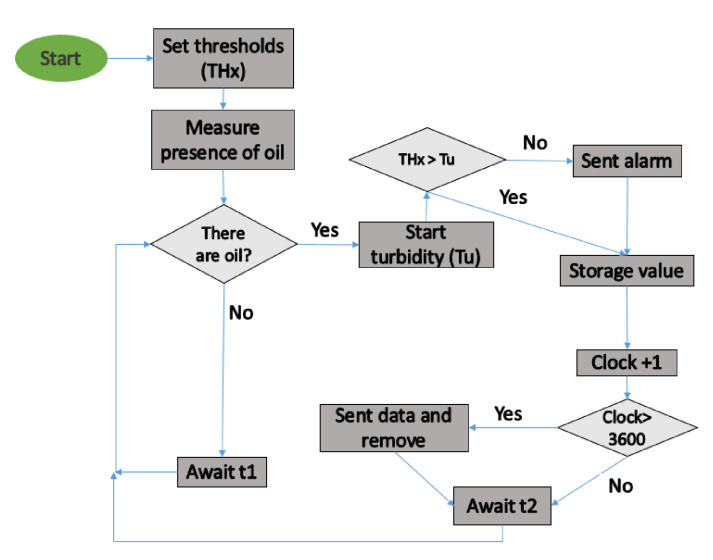
Algorithm of oil detection.

The operational algorithm of the proposed sensor is outlined in Figure 3. First, the threshold values are set (*THx*). Then, the sensor determines the presence of oil in the water (by turning on the LEDs and gathering the data with the LDR). Since the height of the water column might affect the measurement of oil concentration, we use the sensor level presented previously [23]. If the sensor detects oil presence, the turbidity sensor [24] is activated. The turbidity sensor is used to impede false positives of oil presence caused by the presence of particles in the water. To properly control the presence of oil, the measured turbidity value has to be below the pre-established threshold. If turbidity values are below the threshold, the system determines that there is pollution by oil, sends an alarm, and the concentration and type of oil data are stored in the SD card of the node. If the turbidity value is above the threshold, it can be a false positive. In this case, data are stored, but no alarm is triggered. We have limited the number of records that the node can store to 3600. When new data is saved, the algorithm adds +1 to a variable (clock). If the “clock” is full (3600 records stored), the system sends all the stored data and erases the SD to release the internal memory. Then, for starting a new measure, the system waits for a time (t2). In future work, we will use machine learning to obtain an autonomous system able to distinguish between the different oil classes.

## 4. Test Bench

This section shows the used methodology to power the sensor, the obtention of oil, and prepare the samples for calibration and verification tests.

### 4.1. Electronic Operation

An AC power supply model FAC-662B [25] is used to power the LEDs with a voltage of 5 V. The required resistors for each LED are calculated using the voltage drop of the LEDs. We calculated the resistance for an intensity of 15 mA. We selected resistors based on the most similar available standard electronic components. The following resistors, 220 kΩ, 220 kΩ, 150 kΩ, 150 kΩ, and 100 kΩ, were selected for yellow, red, blue, green, and white LEDs respectively. Due to the differences in resistance between the calculated and used resistors, we had different intensities for the LEDs. The intensities were 13.48 mA, 13.00 mA, 12.33 mA, 13.60 mA, and 12.39 mA for the yellow, red, blue, green, and white LEDs. To gather the data, we measured the resistance of the LDRs with a tester (Tenma 72-2600 [26]). As the LDRs have a delay, we waited for 20 s to gather the data for each LED. We repeated the measurements three times in order to work with mean values. Once we gathered the data, we turned off the LED. Then, we turned on the subsequent LED. The sequential order to power the LEDs were yellow, red, blue, green, and white.

### 4.2. Sources of Oil

The industrial fuels that we used in this paper were obtained from a mechanical workshop. Two types of oil were used for the experiment, oil used by a gasoline engine and oil used by a diesel. The oil used was 0w/30, characterised by an SAE (Society of Automotive Engineers) viscosity of 0 in cold conditions and an SAE viscosity of 30 at working temperature. The used oil is from a mechanical workshop of Valencia province (Spain). The oil is INEO FIRST 0W-30 manufactured by TOTAL QUARTZ (Madrid (Spain)). The two oils have similar values of kinematic viscosity with a value of 25.4 mm^2^/s at 25 °C. Furthermore, the oils used belong to different types of engines. The first was used in a diesel engine with 77,000 km and three years of use. A gasoline engine with 31,000 km and two years of use was selected for the second oil source. This type of oil is one of the most common oils used in vehicles and machines. In general, the oil of diesel engines is dirtier than the oil of gasoline engines. Diesel engines generate more particles than gasoline engines, and some end up in engine oil. For this reason, used oils in diesel engines tend to be darker than used oils in gasoline engines. In addition, the used oil of diesel or gasoline engines might present some differences in the additives, neutralization number, oxidation, and nitration [27].

### 4.3. Samples for Calibration and Verification

**Figure 4:**
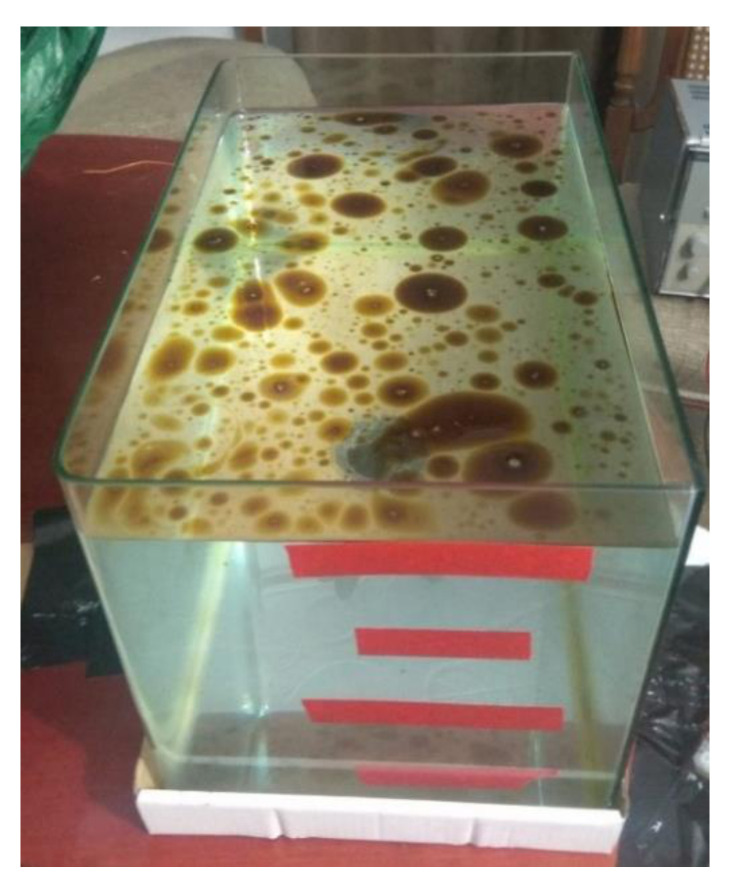
Experimental tank with 0.03 mL_oil_/cm^2^ from gasoline engine at 20 cm height.

The experiment was performed at static conditions introducing the samples in a glass tank of approximately 17 L, measuring 35 cm long, 21 cm wide, and 23 cm high. Although the conditions were static, we noticed that the oil stains had some movement on the water’s surface due to small performances, such as vibrations, that occurred during the measurement. In future work, we will test the sensor under dynamic conditions. The glass tank was marked with red tape at water heights of 0 cm, 5 cm, 10 cm, 15 cm, and 20 cm to carry out the measurements. We gathered the data with water at each one of the different heights. In addition, we obtained the data by setting the LDR to 0° and 180°. Figure 4 illustrates the used tank, with water up to 20 cm and 0.03 mL_oil_/cm^2^ (used by a gasoline engine). In the first round, different amounts of oil were used: 0, 7.1, 21.2, 28.3, 35.4, 56.6, 70.7, 106.1, and 141.5 mL. The used volumes correspond to concentrations of 0, 0.01, 0.03, 0.05, 0.08, 0.1, 0.15, and 0.2 mL on the surface of the water. Each of these oil quantities was measured at different water heights. The data obtained was then used to obtain a calibration. Once the calibration had been obtained, four new samples were used to verify the models obtained. The samples for the verification fad the following characteristics: 0.02, 0.06, 0.12, and 0.18 mL_oil_/cm^2^.

## 5. Results

In this section, the results obtained by our sensor are described. First, we analyze the performance of the developed sensor with industrial oil from a diesel engine. Then, we display the results with the industrial oil from a gasoline engine. Finally, the verification of the obtained mathematical models is analyzed.

### 5.1. Industrial Oil of Diesel Engine

This subsection shows the sensor’s performance when the pollution comes from industrial oil used by a diesel engine.

**Figure 5:**
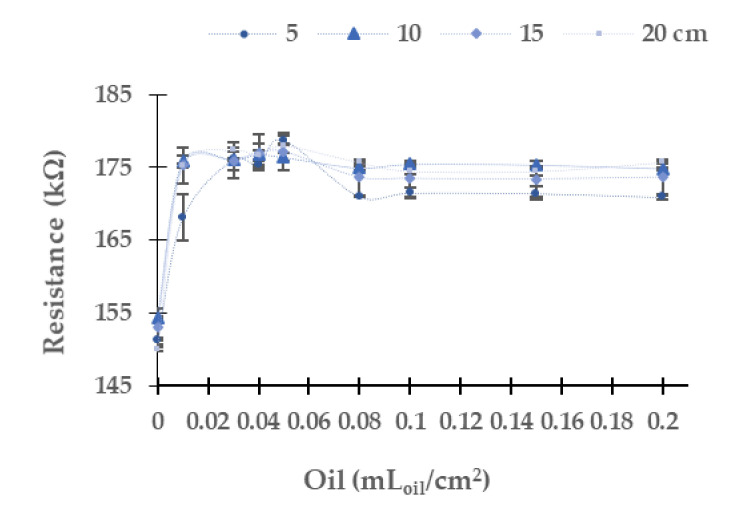
Resistance of LDR at 0° in diesel oil in the yellow LED.

**Figure 6:**
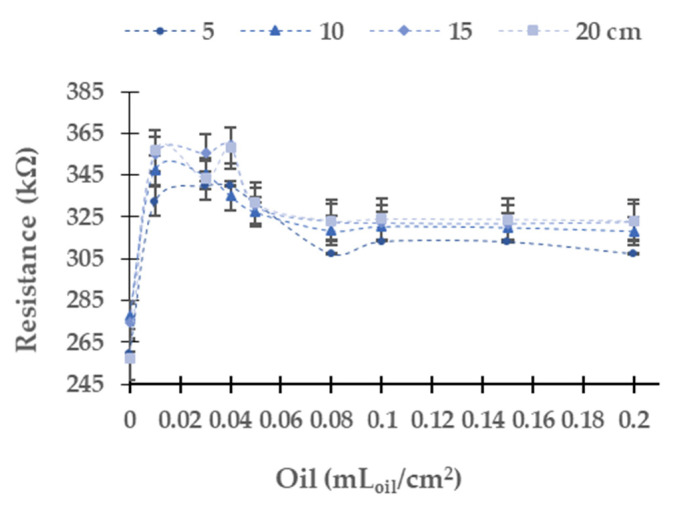
Resistance of the LDR at 0° in diesel oil in the red LED.

**Figure 7:**
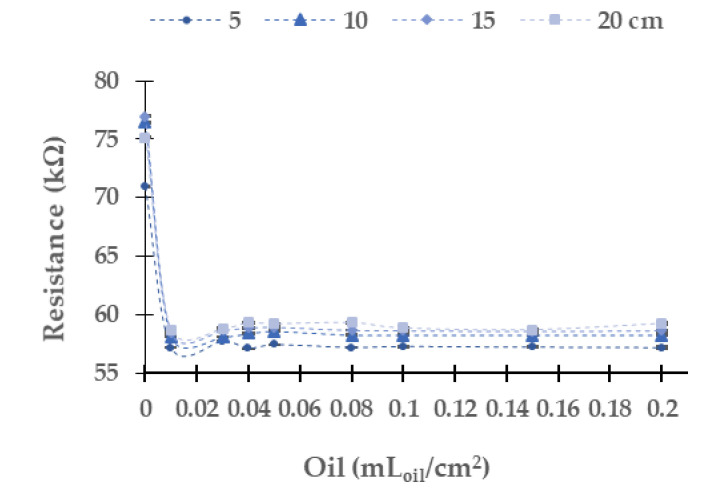
Resistance of the LDR at 0° in diesel oil in the blue LED.

**Figure 8:**
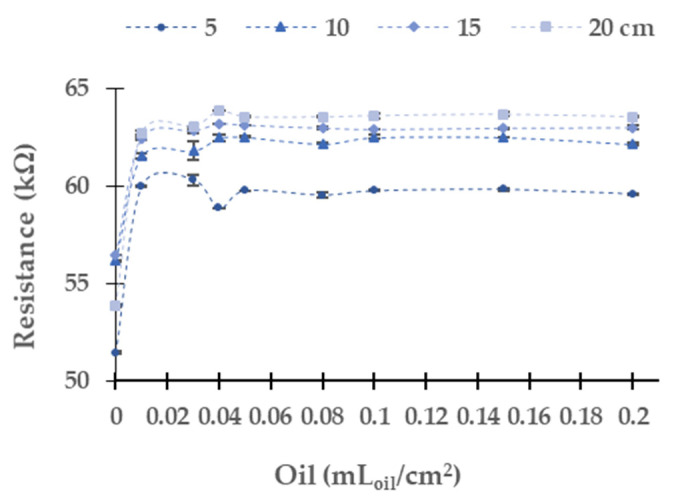
Resistance of the LDR at 0° in diesel oil in green LED.

**Figure 9:**
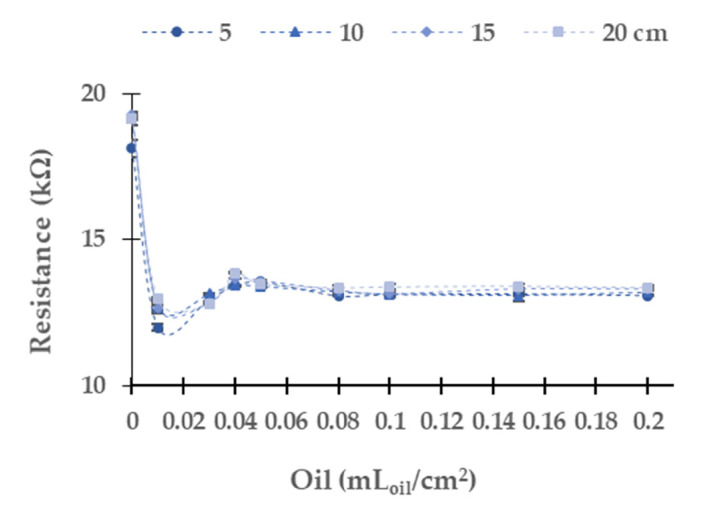
Resistance of the LDR at 0° in diesel oil in white LED.

Figure 5, Figure 6, Figure 7, Figure 8 and Figure 9 represent the data of the LDR’s resistance at 0° for the different light sources tested. We verify that the resistances of the LDR at 180° are not stable due to oil stains moving on the water surface, which causes shadows on the 180° LDR. It is expected that in real conditions, this situation gets worse. Therefore, we decided not to use this LDR. In general terms, and before analyzing case per case, we can identify two different behaviours according to the gathered data. On the one hand, we can see that the resistance of the LDR increases when the concentration of samples increases. This phenomenon indicates that the oil in the water layer absorbs more photons than the same portion of the interface water/air (in the samples without oil). Thus, fewer photons are reflected and reach the LDR, increasing its resistance value compared with the samples without oil. On the other hand, we can find that the oil layer reflects more photons than the interface water/air. Thus, more photons will reach the LDR, decreasing its resistance. The chemical compounds’ optical behaviour explains the different tendencies in the oil and the wavelength of the different light sources.

In the following, we analyze the resistance values of the LDR with different light sources and water column heights. The LDR resistance values for the different concentrations of oil used by a diesel engine when the yellow light source is used are displayed in Figure 5. The resistance values of LDR of the four tested heights in the concentration of 0 mL_oil_/cm^2^ are around 151 kΩ. The resistance of the LDR increases when the oil concentration rises. The absolute (and relative) differences between the water without oil and the maximum oil concentration tested are 19.6 kΩ (12.94%) at 5 cm, 20.5 kΩ (13.26%) at 10 cm, 20.6 kΩ (13.44%) at 15 cm, and 25.7 kΩ (17.15%) at 20 cm. For the four tested heights, the maximum value of resistance is found at 0.05 mL_oil_/cm^2^. From this concentration, the resistance values decrease slightly (except for 5 cm height, where there is a significant decrease in resistance of the LDR between concentrations of 0.05 and 0.08 mL_oil_/cm^2^). The resistance values obtained with a water column of 5 cm are slightly away from the rest of the heights for an identical oil concentration. Meanwhile, the values of 10, 15, and 20 cm are relatively similar. Regarding the dispersion of the results in general, this is low. The relative average of standard dispersion is 0.30%, and the maximum standard dispersion occurs in the case of 0.01 mL_oil_/cm^2^ at 5 cm of water with a value of 3.23 kΩ.

The data gathered using the red LED as a light source is represented in Figure 6. The red light has the maximum absolute resistance difference in the LDR between the water without oil and the maximum concentration tested among all tested light. The absolute (and relative) resistance difference between the minimum and maximum oil tested is 48.1 kΩ (18.5%) at 5 cm, 40.3 kΩ (14.5%) at 10 cm, 48.2 kΩ (17.6%) at 15 cm, and 66.3 kΩ (25.8%) at 20 cm. As in the yellow light source, at all tested heights, LDR resistance values present a great change between 0 to 0.01 mL_oil_/cm^2^. The resistance values are similar in each height between the concentration of 0.01 and 0.4 mL_oil_/cm^2^. At all heights, when the oil concentration increases to 0.05 mL_oil_/cm^2^, the resistance data upsurges. At 5 cm, we observe a significant decrease of the resistance value of the LDR for the samples with concentrations from 0.05 to 0.08 mL_oil_/cm^2^. At this height, with the increase of oil concentration, the values are stable. The samples with a concentration above 0.05 mL_oil_/cm^2^ present similar values in the other heights.

In this light source, there is a significant dispersion of the resistance values in the LDR. The maximum standard deviation is 6.99 kΩ. The average standard deviation is 0.84 kΩ, and the relative average standard deviation is 0.25%.

Figure 7 presents the results gathered when the blue light is used. The absolute (and relative) resistance difference in the LDR between the concentrations of 0.00 to 0.20 mL_oil_/cm^2^ is 13.8 kΩ (19.4%) at 5 cm, 18.2 kΩ (23.8%) at 10 cm, 18.3 kΩ (23.8%) at 15 cm, and 15.9 kΩ (21.2%) at 20 cm. In contradistinction to the results above, the resistance values of LDR decrease with the increasing oil concentration. In this light, the dispersion of resistance values is low; both maximum and average values are low. The maximum standard deviation is 0.16 in 0.01 mL_oil_/cm^2^ at 15 cm, and the relative average standard deviation is 0.091%. As in the other light sources previously studied, the values gathered at 5 cm differ from the other resistance values gathered at different water column heights. In this case, we do not observe a tendency such as in yellow and red LEDs. The main change of resistance is between 0 to 0.01 mL_oil_/cm^2^. Then, from 0.03 mL_oil_/cm^2^ to 0.20 mL_oil_/cm^2^, the resistance values are similar, tending to 57 kΩ.

Figure 8 displays the data gathered (LDR at 0°) when the green light is used. The absolute (and relative) differences between the first and last samples are 8.1 kΩ (15.8%) at 5 cm, 6.0 kΩ (10.6%) at 10 cm, 6.5 kΩ (18.0%) at 15 cm, and 9.7 kΩ (18.0%) at 20 cm. The values of resistance tend to an upper bound of 60 kΩ at 5 cm, 62.13 kΩ at 10 cm, 62.93 kΩ at 15 cm, and 63.53 kΩ. The maximum dispersion is 0.49 kΩ in the concentration of 0.15 mL_oil_/cm^2^, and the average dispersion is 0.07 kΩ.

The resistance values measured with the LDR when the white LED is selected as a light source are presented in Figure 9. In this case, the absolute (and relative) differences are 5.0 kΩ (27.8%) at 5 cm, 5.9 kΩ (31.1%) at 10 cm, 6.0 kΩ (31.1%) at 15 cm, and 5.8 kΩ (30.3%) at 20 cm. Except in the concentration of 0 and 0.01 mL_oil_/cm^2^, the rest of the resistance values for the different heights are similar. In this case, the maximum standard deviation and average for the concentration of 0.01 mL_oil_/cm^2^ at 20 cm are 0.34 kΩ and 0.06 kΩ, respectively.

Subsequently, we apply a multifactorial Multivariate Analysis of Variance (ANOVA) using Statgraphics Centurion XVI [28] to analyze if the observed differences between resistance values at different concentrations and different heights are significant or not. The results for the analyses are outlined in Table 1. In all the cases, the *p*-value is lower than 0.05. Thus, we can state with a probability of 95% that there are statistical differences in the different heights. We do the same for the oil used by an engine and verify that the value of *p* is lower than 0.0001.

Although there is a statistical difference among the LDR resistance values for all the used light sources, the lack of a homogeneous trend along the tested samples precludes determining the sample’s concentration. Thus, it is impossible to have a calibration for detecting industrial oil used by diesel engines. The sole option is to determine the presence or absence of oil.

### 5.2. Industrial Oil of Gasoline Engine

**Figure 10:**
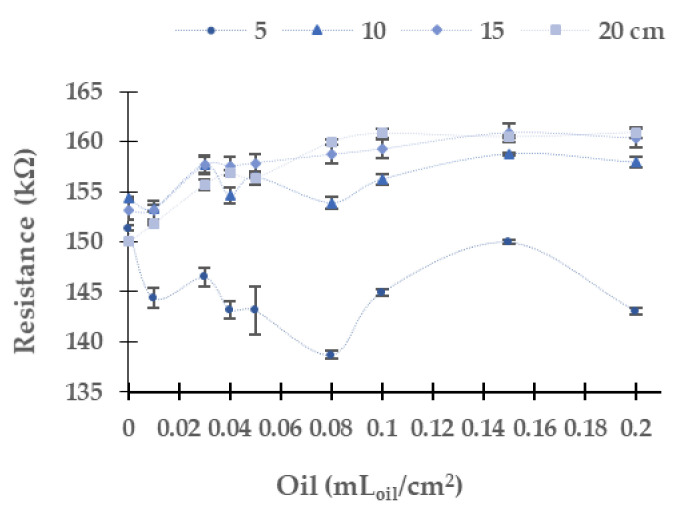
Resistance of the LDR at 0° in gasoline oil in the yellow LED.

In this subsection, we analyze the resistance values of the LDRs obtained with different light sources and using the industrial oil used in the gasoline engine. As in the case of oil used by a diesel engine, we discard the use of LDR at 180° due to the oil stains generated shadows in the LDR. Now, we focus on the change of resistance of LDR with the different colour light sources and oil concentrations. First, we analyze the results gathered using the yellow light, which are displayed in Figure 10. The absolute (and relative) difference in resistance values between the maximum and minimum concentration of oil is 3.5 kΩ (3.5%) at 10 cm, 7.2 kΩ (7.2%) at 15 cm, and 10.9 kΩ (10.9%) at 20 cm. At the height of 5 cm, the resistance values do not have a homogeneous and defined trend. The most probable explanation for the gathered data is the non-homogeneous distribution of the oil over the water. This phenomenon is observed to a lesser extent in industrial diesel engine oil. At 10 cm of the water column, there is no evident trend in the LDR’s resistance values for concentrations from 0 to 0.08 mL_oil_/cm^2^. Instead, we detect continuous growth at 15 cm and 20 cm until reaching an upper limit, approximately 161 kΩ. When the yellow light is used, we observe few dispersion data. The maximum standard deviation is 2.38 kΩ with an average of 0.48 kΩ, and a relative standard deviation of 0.31%.

**Figure 11:**
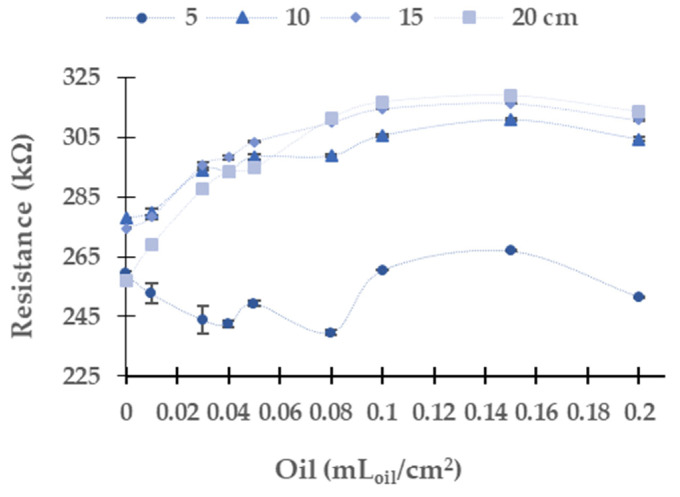
Resistance of the LDR at 0° in gasoline oil in red LED.

Figure 11 represents the LDR resistance values for the red light using the oil of the gasoline engine. The absolute (and relative) difference in resistances values are 26.3 kΩ (9.5%) at 10 cm, 36.1 kΩ (13.2%) at 15 cm, and 56.5 kΩ (56.5%) at 20 cm between the water without oil and the maximum concentration of oil. For 5 cm of the water column, the resistance values do not have a defined tend. We observe that the values measured at 10 cm, 15 cm, and 20 cm tend to grow when oil concentration increases. The maximum standard deviation is found for the height of 5 cm. The standard deviation is generally low, with an average standard deviation of 0.67 kΩ with all data gathered. Since data follow a homogeneous trend (except for the last two samples) and the standard deviation is low, we can use these values for calibration.

(1) $$Oil\;\left(\frac{{\mathrm{mL}}_{\mathrm{oil}}\;}{{\mathrm{cm}}^{2}}\right)={\left(-2.78924+0.0101666\times \mathrm{Resistance}\;\left(k\Omega \right)\right)}^{2}$$

(2) $$Oil\;\left(\frac{{\mathrm{mL}}_{\mathrm{oil}}}{{\mathrm{cm}}^{2}}\right)={\left(-1.88586+0.00698826\times \mathrm{Resistance}\;\left(k\Omega \right)\right)}^{2}$$

(3) $$Oil\;\left(\frac{{\mathrm{mL}}_{\mathrm{oil}}}{{\mathrm{cm}}^{2}}\right)={\left(-1.26118+0.00497868\times \mathrm{Resistance}\;\left(k\Omega \right)\right)}^{2}$$

**Figure 12:**
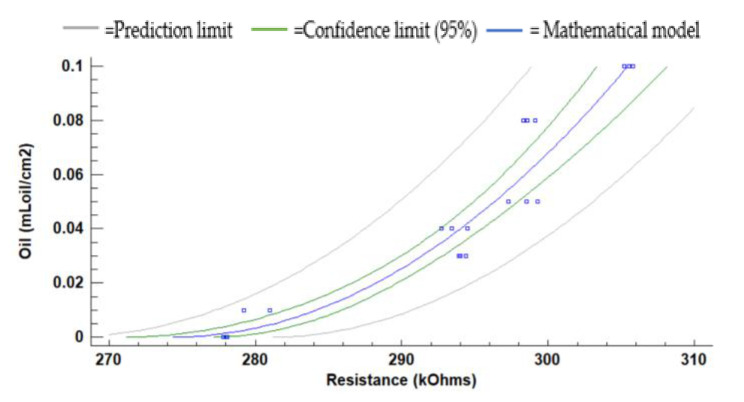
The mathematical model for the red light at 10 cm to oil using the gasoline engine.

**Figure 13:**
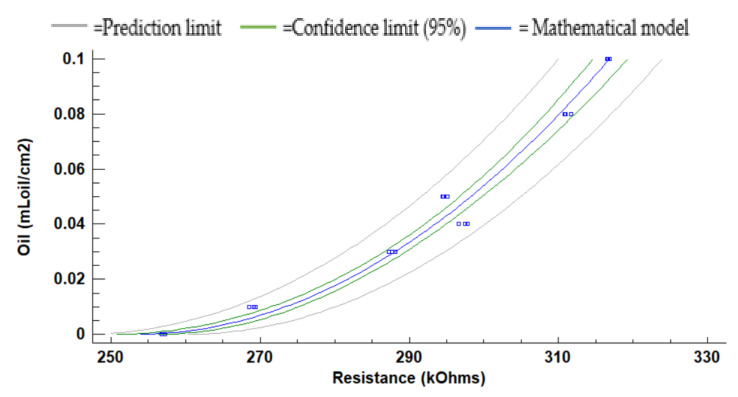
The mathematical model for the red LED at 15 cm to oil using the gasoline engine.

**Figure 14:**
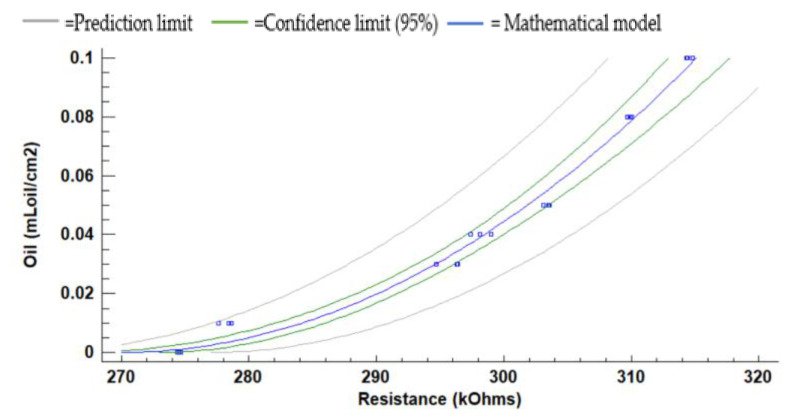
The mathematical model for the red LED at 20 cm to oil using the gasoline engine.

The calibration models, including the confidence and prediction limits obtained for the red light at 10, 15, and 20 cm, are presented in Figure 12, Figure 13 and Figure 14. Note that the data gathered with the two samples with the highest concentrations are not used for the calibration since they do not follow the same trend as the rest of the data. Mathematical models for the heights at 10 cm, 15 cm, and 20 cm are calculated with Statgraphics Centurion XVI, obtaining the following correlations R2 0.9138, 0.9560, and 0.9784. The mathematical models are presented in Equations (1)–(3) for 10 cm, 15 cm, and 20 cm. The average Absolute Errors (AEs) are 0.0074 mL_oil_/cm^2^ for 10 cm, 0.0031 mL_oil_/cm^2^ for 15 cm and 0.0023 mL_oil_/cm^2^ for 20 cm. The Relative Errors (REs) are 25.9% for 10 cm, 15.8% for 15 cm and 10.2% for 20 cm.

**Figure 15:**
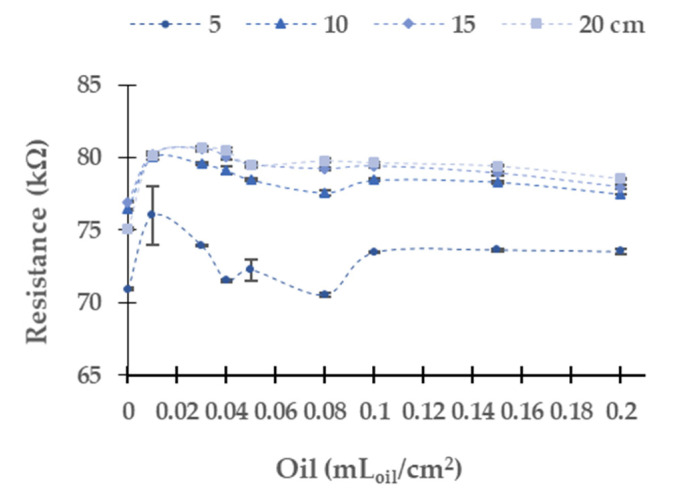
Resistance in LDR at 0° in gasoline oil in blue LED.

In the following, we present in Figure 15 the measured resistances in the LDR using blue light. The absolute (and relative) differences in resistances between the samples are 1.1 kΩ (1.4%) at 10 cm, 1.0 kΩ (1.3%) at 15 cm, and 1.0 kΩ (4.6%) at 20 cm. These low differences preclude using this LED to monitor the presence of oil in a gasoline engine. As in the previous cases for oil used by a gasoline engine, the height of 5 cm does not have a defined tendency. The rest of the water column heights presents an increase of the resistance values between 0 to 0.01 mL_oil_/cm^2^. From this point, a general reduction of resistance is produced. The resistance values at 10 cm are lower than at 15 cm and 20 cm of water column heights. The average standard deviation is 0.25 kΩ.

**Figure 16:**
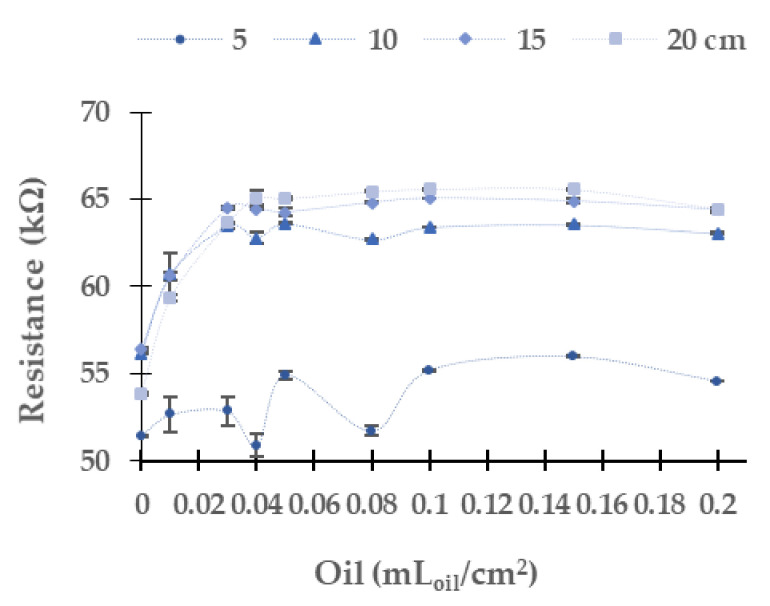
Resistance in LDR at 0° in gasoline oil in green LED.

Figure 16 represents the results of the LDR using the green light. The absolute (and relative) differences in resistance values are 6.9 kΩ (12.3%) at 10 cm, 7.9 kΩ (14.1%) at 15 cm, and 10.6 kΩ (19.7%) at 20 cm. In this case, the values tend to grow between 0 and 0.04 mL_oil_/cm^2^. From this concentration, resistance values are close to an upper limit. The average deviation standard is 0.19 kΩ with a maximum value of 1.20 kΩ.

**Figure 17:**
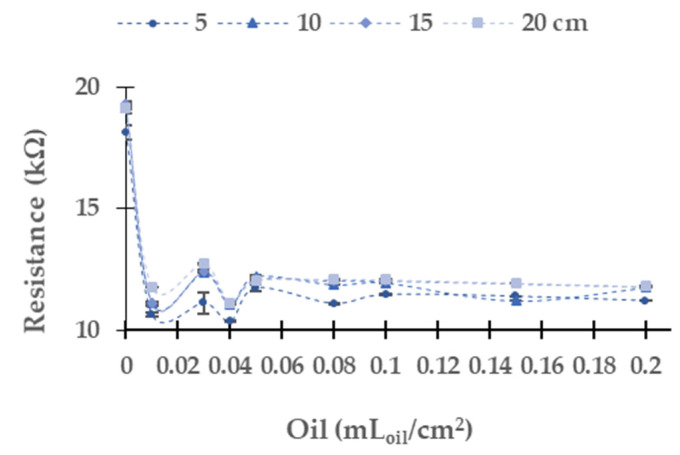
Resistance in LDR at 0° in gasoline oil in white LED.

Finally, Figure 17 displays the resistance values of the LDR obtained using white light. In this case, we identify a similar trend than in the blue LED with the diesel oil. The tendency, opposed to observed ones in the rest of the LEDs, is a decreasing resistance when the concentration increases. The absolute (and relative resistances) differences in resistances between the samples are 6.9 kΩ (38.6%), 7.9 kΩ (38.9%), and 10.6 kΩ (38.5%) at 10 cm, 15 cm, and 20 cm respectively. The change in the resistance occurs between the concentrations of 0 and 0.01 mL_oil_/cm^2^. However, at 0.03 mL_oil_/cm^2^, there is a slight increase in resistance values. At 0.04 mL_oil_/cm^2^, there is a decrease in the LDR resistance at all the measured heights. Then from 0.05 mL_oil_/cm^2^, there is a gradual decrease in resistance values. The average standard deviation is 0.06 kΩ. This deviation is the lowest one among all the LEDs used for monitoring the presence of oil used by a gasoline engine.

We apply a multifactorial ANOVA to analyze if the observed differences between resistance values at different concentrations and different heights are significant or not, as in the previous subsection. The results for the analyses are outlined in Table 2. The results indicate that both the height and oil concentration have statistically significant effects on the value of LDR resistance. There is an exception in the case of the yellow LED, for which the concentration of oil has no effect.

### 5.3. Verification

In this subsection, we verify the function of our prototype by measuring four samples, the concentrations of which are different to the ones used in the calibration.

As we said before, concerning the oil from diesel engines, we can differentiate between the presence or absence of an oil layer but not quantify it. Thus, a verification of a proper mathematical model is not possible.

Concerning oil used in a gasoline engine, the red light is the one that presents an optimal behaviour to determine the concentration of oil used by a gasoline engine. Previously, in the red light, we discard the height of 5 cm of the water column due to its high dispersion of data and its non-regular trend. We verify the mathematical models of the heights 10, 15, and 20 cm (Equations (1)–(3)). The results obtained are shown in Table 3. Previously, we decided to use 4 verification points. Nonetheless, the concentration of 0.18 mL_oil_/cm^2^ was discarded because this concentration was higher than the ones used for the calibration of the mathematical model. Moreover, the point 0.12 mL_oil_/cm^2^ is a higher oil concentration than the calibration points. However, it is a value close to those used to calibrate the model and is included.

Regarding the verification results, the average RE is 19.9% at 10 cm, 13.7% at 15 cm and 11.1% at 20 cm. At the 15 cm and 20 cm heights, the maximum RE is found in the concentration of 0.06 mL_oil_/cm^2^ with values of 30.9% at 15 cm and 21.3% at 20 cm. Regarding the verification, at 10 cm the maximum RE is 27.1% at 0.02 mL_oil_/cm^2^.

## 6. Discussion

This section discusses the obtained results. First, we analyze the results obtained for the two sources of pollution. Next, we present the algorithm to calculate oil concentration in water. Finally, we discuss the lessons learned and the limitations of our study. Finally, the relevance of this sensor for smart irrigation is argued for.

### 6.1. Differences among Oils

In this subsection, we analyze the differences among the obtained results with the different pollutants. Table 4 outlines the minimum and maximum resistance values gathered with the two oils (gasoline and diesel engine). LDR combined with green and white lights present similar minimum and maximum resistance values. Meanwhile, when combined with yellow, red, and blue lights, they present higher resistance differences with the two tested oils.

Summarizing the obtained values of the two experiments, we can identify some differences, which can be used to create our sensor. For example, the red LED can be used as a light source to quantify the volume of contamination in the oil used by gasoline engines, while no one LED can be used to quantify the oil used by diesel engines. We observe an important difference in the trends by comparing the resistance values with oil from a gasoline engine with the same oil from a diesel engine using the blue LED. There is a rapid reduction of the resistance values in diesel oil, which is not identified with the oil used by a gasoline engine. This fact can be used for determining the presence of oil used by gasoline or diesel engines. Thus, we can affirm that there is an option to combine different LEDs to create a sensor for industrial oil detection and quantification in certain cases.

### 6.2. Selection of LEDs

In this subsection, we present in detail the best configuration of LEDs and the operation algorithm for the developed sensor.

After checking the results, we can affirm that quantifying the oil concentration used in diesel engines in water is not possible with our prototype. Nonetheless, it is possible to determine the presence or absence of oil used in diesel engines. The green, white, and blue LEDs can be used for that purpose. They all present a great difference of resistance between the concentration of 0 to 0.01 mL_oil_/cm^2^, and have similar resistance values in concentrations higher than 0.01 mL_oil_/cm^2^. Since the white LED has the highest relative difference between water with and without oil, it is the best option to determine the presence of oil. To determine the source of oil, we use the blue LED. The resistance value of the LDR is different according to the origin of oil present in the water. If the oil is from a gasoline engine, the red LED can be used to determine the concentration. Next, we are going to detail the steps followed to determine the presence of oil in water followed by our prototype.

The first step in our operational algorithm is to determine if there is oil in the water and to identify if the oil comes from a diesel or gasoline engine. We use the white LED to determine the presence or absence of oil in water. The minimum resistance of the LDR with the white LED when there is no oil is 18.2 kΩ. Meanwhile, when oil concentration is 0.01 mL_oil_/cm^2^ or higher, the resistance value decreases to 12 kΩ or 11 kΩ, according to the origin of the oil, diesel, or gasoline engine. Therefore, if the measured resistance value on the LDR is lower than 18 kΩ, we can assume that there is oil pollution in the water. Once the presence of oil in the water has been determined, the next step is to ascertain whether the oil comes from a diesel or gasoline engine. We use the blue LED to recognize the origin of the oil. In gasoline oil, the resistance of the LDR increases with the concentration of oil. On the contrary, if the oil was used by a diesel engine, the resistance decreases with the concentration of oil. On the one hand, with the blue light, the resistance values of LDR when the origin of oil is a diesel engine are below 60 kΩ. On the other hand, the resistance values are close to 75 kΩ (or higher) when the oil was used by a gasoline engine. Thus, we set a threshold of 70 kΩ to determine the origin of the oil.

Regarding the oil of a gasoline engine, it is possible to quantify its concentration using the red LED. Nevertheless, in this case, the height has a significant impact on the gathered data. Therefore, the water height must be measured in order to select which calibration model must be applied (see Equations (1)–(3)). If the water column height is lower than 7.5 cm, we cannot apply any of the equations, since we are reaching the scenario with a water column equal to 5 cm and our results indicate that it is not possible to have a calibration. If the water column is higher than 22.5 cm, we cannot use any equation because the height of the water is far away from the highest tested water height and no information about the expected results is obtained. In these two cases, the algorithm can indicate the origin of the oil but not its concentration. For water column heights between 7.5 and 22.5 cm, interpolation will be carried out between Equations (1)–(3), depending on the case.

**Figure 18:**
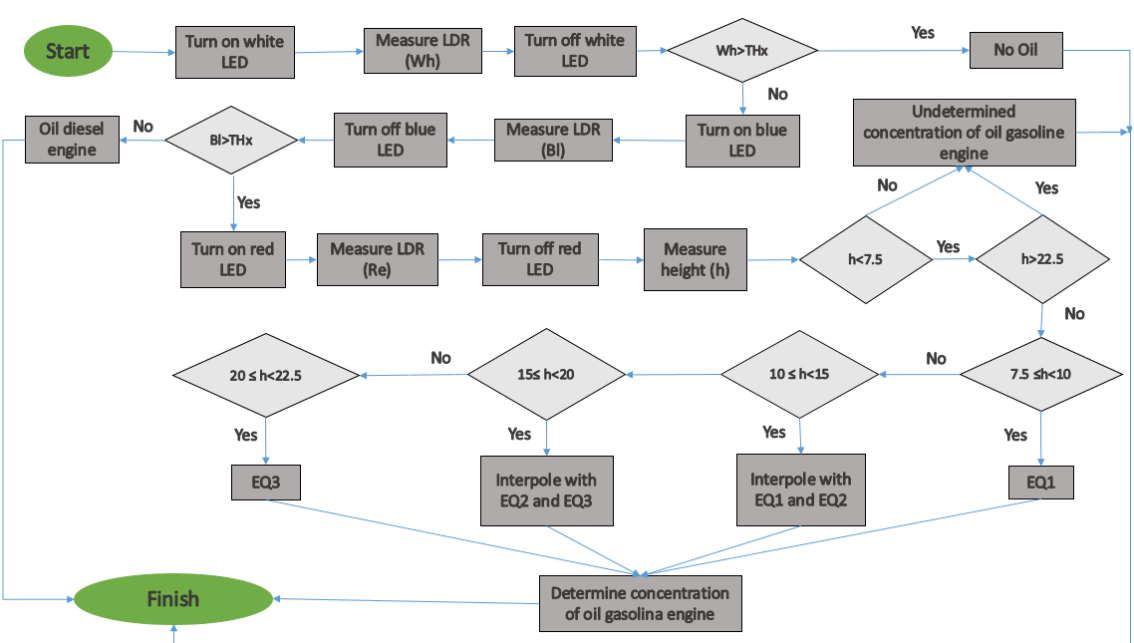
Algorithm of the function of our sensor.

To sum up, we use a combination of LEDs in our prototype. The white LED for determining the presence of oil, the blue LED to identify the source of the oil. The red LED is used to quantify the oil concentration if it comes from a gasoline engine in water heights between 10 to 22.5 cm. The whole process with the thresholds can be seen in Figure 18.

### 6.3. Lessons Learned and Limitations of the Present Study

Along with this study, we have identified some interesting issues which must be considered for future sensors for water quality designs. First and foremost, the irregular dispersion of oils in the water surface causes a series of shadows and irregularities in the light distribution, which precludes the use of the light receptor at 180° from the light source. Moreover, this dispersion also causes many difficulties when the sensor (at 0° from the light source) is placed too close to the water surface, at 5 cm or less. Thus, in future designs and test benches, we will establish the sensors farther from the water surface. We tested the sensor in static conditions. In the future, we will test in a real irrigation channel. Sloshing from the oil and water interface can lead to problems for oil determination. To solve this problem, the sensor will be located in areas with a laminar water regime. Moreover, the inclusion and the most appropriate orientation and materials of measuring conduct to avoid bubbles, voluminous suspended solids, and fouling will be studied. In addition, in future work, we will propose the use of artificial intelligence.

Concerning the different pollution sources used in this study, we have a limitation that should be solved in future work; we have not used industrial oil from agricultural machinery. Nonetheless, most agroindustrial machinery uses diesel, and therefore we expect similar results to those in the diesel source used in this paper. It must be noted that this machinery uses ‘B’ diesel, which includes dyes to avoid its use in regular vehicles. Therefore, the effect or the transference of these dyes to the oil must be studied.

Regarding some possible interferences of other pollution sources, the combination of the turbidity sensor with the proposed prototype reduces the range of pollutants, which can produce a false negative. Despite this, other organic compounds with density values lower than the water might interfere with our sensor. Some examples are gasoline and diesel. The water velocity might also reduce the accuracy of our sensor. If the water current is too high, it will move and disperse the pollution very fast, which will make reading the resistance of the LRD or precluding the competition of the entire operational algorithm before the water characteristics change difficult.

### 6.4. Relevance of the Present Study for Smart Irrigation and SMARTWATIR Project

Although the pollution with industrial oil of irrigation channels is a relatively controlled issue in developed countries, it is not a controlled issue in developing countries. As far as we know, there is no evidence in papers or reports that reviews the periodicity of pollution events linked to industrial oil of agricultural machinery. Nonetheless, the impact of this pollution is very high, affecting the soils, the aquifers and even the yield, as set out in the introduction. One of the principal reasons for this lack of information is the fast dispersion and local incidence jointly to the vast surface of irrigation channels, which difficulties the data sampling and monitoring. Therefore, the inclusion of this low-cost sensor will be crucial in monitoring pollution in irrigation channels. This is particularly important in scenarios where smart irrigation is being applied, since the industrial oil can produce interferences in other sensors (such as conductivity sensors) or foul other optical sensors placed on the water surface.

Focusing on the SMARTWATIR project and the phytodepuration, the detection of pollutants such as industrial oil must be accomplished before the phytodepuration in order to determine if the water can be regenerated or not. Moreover, some pollutants might be toxic for the plants, jeopardizing the entire ecosystem.

## 7. Conclusions

In this paper, we evaluated the application of an optical sensor to measure industrial oil concentration in irrigation water. For this purpose, we used an assay which included two types of industrial oil (from a diesel engine and a gasoline engine). We evaluated the sensor’s performance at four different water heights (5, 10, 15, and 20 cm). As a light source, we used yellow, red, blue, green, and white LEDs.

The white LED can be used to detect the absence or presence of industrial oil. Meanwhile, the blue LED can determine the source of this industrial oil. For pollution coming from the oil used by a diesel engine, at all light sources and water column heights, the LDR resistance has an abrupt change between 0 to 0.01 mL_oil_/cm^2^, allowing the detection of pollution. At higher concentrations, the values approached an upper limit or decreased slightly, precluding the determination of oil concentration. For the oil used by a gasoline engine, the red light is the unique one that presents homogeneous changes in the LDR’s resistance values at all tested concentrations, allowing us to measure the concentration of the industrial oil. The calibration verification provided an average RE of 19.9%, 13.7%, and 11.1% for 10, 15, and 20 cm, respectively. Thus, our prototype will combine different light sources for monitoring oil in irrigation water.

In future work, experiments will be carried out by making measurements under real conditions, by using a dynamic scenario to test the developed sensor. In this way, we will evaluate the quantification capacity of the oil in an irrigation system. Furthermore, measurements will be made by varying the turbidity of the water to simulate possible situations in the irrigation canal. On the other hand, we will incorporate oil used by farming machinery and test the sensor’s performance at higher water column heights. Finally, a photodiode will be incorporated into the sensor to take measurements at longer wavelengths, such as infrared, and to optimize the sensor’s operation.

## Figures and Tables

**Table 1:** Multivariate analysis of variance for the height and concentration of diesel oil.

	*p*-Value for Height	*p*-Value for Concentration
Yellow	0.0031 ***	<0.0001 ***
Red	0.0006 ***	<0.0001 ***
Blue	<0.0001 ***	<0.0001 ***
Green	<0.0001 ***	<0.0001 ***
White	0.0107 ***	<0.0001 ***

Significance levels: *** *p* < 0.05.

**Table 2:** Multivariate analysis of variance for the height and concentration of gasoline oil.

	*p*-Value for Height	*p*-Value for Concentration
Yellow	<0.0001 ***	0.0977
Red	0.0003 ***	<0.0001 ***
Blue	<0.0001 ***	<0.0001 ***
Green	<0.0001 ***	<0.0001 ***
White	<0.0001 ***	<0.0001 ***

Significance levels: *** *p* < 0.05.

**Table 3:** Verification for the red LED.

Height (cm)	Concentration (mL_oil_/cm^2^)	Calculated Concentration (mL_oil_/cm^2^)	AE (mL_oil_/cm^2^)	RE (%)
10	0.02	0.025	0.005	27.1
10	0.06	0.073	0.013	21.1
10	0.12	0.134	0.014	11.5
15	0.02	0.020	0.000	2.3
15	0.06	0.079	0.019	30.9
15	0.12	0.111	0.009	7.9
20	0.02	0.019	0.001	2.7
20	0.06	0.073	0.013	22.0
20	0.12	0.110	0.010	8.5

**Table 4:** Maximum and minimum resistance measured when the two oils.

Colour	Gasoline		Diesel	
	Minimum (kΩ)	Maximum (kΩ)	Minimum (kΩ)	Maximum (kΩ)
Yellow	138.68	160.91	150.01	178.81
Red	239.5	318.83	256.97	359.19
Blue	70.56	80.66	57.09	76.94
Green	50.88	65.57	51.44	63.85
White	10.35	19.29	11.98	19.29

## Data Availability

The data presented in this study are available on request from the corresponding author. The data are not publicly available due to privacy constrains.
